# Tile-based massively scalable MIMO and phased arrays for 5G/B5G-enabled smart skins and reconfigurable intelligent surfaces

**DOI:** 10.1038/s41598-022-06096-9

**Published:** 2022-02-17

**Authors:** Xuanke He, Yepu Cui, Manos M. Tentzeris

**Affiliations:** Georgia Institute of Technology, Electrical and Computer Engineering, Atlanta, 30309 USA

**Keywords:** Engineering, Electrical and electronic engineering

## Abstract

This work presents a novel tile based approach to constructing, in a modular fashion, massively scalable MIMO and phased arrays for 5G/B5G millimeter-wave smart skins and large-area reconfigurable intelligent surfaces for Smart Cities and IoT applications. A proof-of-concept 29 GHz 32 elements phased array utilizing $2 \times 2$ “8-element subarray” tiles was fabricated and measured and demonstrates $+/-$ 30beamsteering capability. The unique benefits of the proposed tile approach utilizes the fact that tiles of identical sizes can be manufactured in large quantities rather than have arrays of multiple sizes serve various user capacity coverage areas. It has to be stressed that the proof-of-concept flexible $2 \times 2$ tile array features no performance degradation when it is wrapped around a 3.5 cm radius curvature. This topology can be easily scaled up to massively large arrays by simply adding more tiles and extending the feeding network on the mounting tiling layer. The tiles are assembled onto a single flexible substrate which interconnects the RF, DC and digital traces, allowing for the easy realization of on-demand very large antenna arrays on virtually any practical conformal platform for frequencies up to sub-THz frequency range.

## Introduction

Recently, the telecommunications industry has been rapidly transitioning to 5G standards for faster, higher capacity and lower latency communications. One of the most crucial requirements for the successful implementation of these 5G and B5G (Beyond 5G) technologies, especially for millimeter-wave (mmWave) and sub-THz frequencies, is the realization of large antenna arrays for massive MIMO configurations^1^. However, these large antenna arrays are typically quite bulky and heavy and come only in very limited sizes, and thus increasing the cost of customization and reducing the adaptability to various end-use cases. For 5G mmWave networks, due to their inherently reduced range, implementations have, shifted to utilizing small/pico cell architectures with each hotspot granting coverage of 50–100 m^2^. The use of small cells means that various locations can vary widely in terms of usage rates, for example a sports stadium versus suburban areas. Therefore there is not a one-size-fits-all approach to 5G/B5G and IoT implementations.

**Figure 1:**
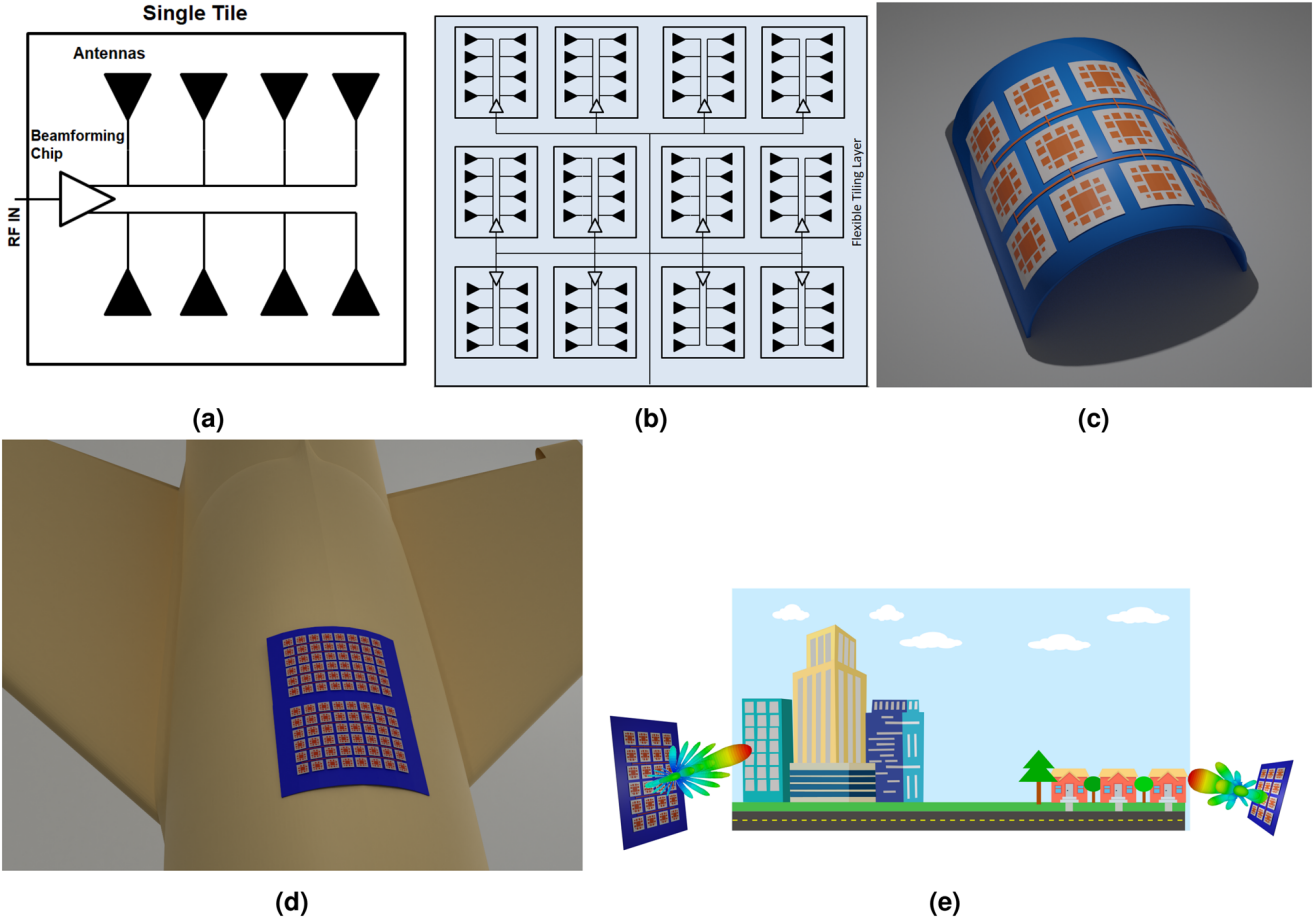
(**a**) Single Tile and (**b**) multi-tile schematic of the proposed massively scalable modular antenna array architecture. (**c**) 3D image showing the tiles placed on a flexible tiling layer which allows it to be conformed onto curved surfaces for very large antenna arrays used in “Smart Skin” implementations, such as the surface of an aircraft (**d**). (**e**) The proposed tile based architecture grants an easy way to scale up or down reconfigurable intelligent surfaces (RIS) and MIMOs for high or low density 5G/B5G coverage areas dramatically reducing the cost and enhancing on-demand modularity and scalability.

The solution proposed in this work is the utilization of antenna array tiling to build phased arrays at mmWave frequencies. A general schematic for this design architecture and applications for this technology is shown in Fig. 1a, b. This type of technology can be utilized into many applications for flexible massive MIMOs, Fig. 1c, Smart-Skin (d), and for on-demand modular and customizable very large phased array applications (e). Various mentions of tile based phased array architectures can be found in literature such as^3–7^. Additionally, antenna arrays featuring removable antennas were discussed in^8,9^. However in^3^ and^4^, the tile based elements were entirely built on a rigid PCB with single antenna element tiles and don’t demonstrate the modularity of the design^5^. Features tiles on a die level, which is difficult to assemble due to the need for packaging. Additionally it is also featured on a rigid substrate. In^7^, a flexible implementation is introduced, however the tiles do not display any modularity as this implementation is a single substrate design. With works such as^8^ and^9^, the modular antenna elements require SMA cabling, that can become easily too cluttered for large arrays. Additionally the need for discrete components adds cost and integration complexity. There have also been developments in utilizing meta-material components such as in^10^ for meta-surfaces as a way to realize massive amounts of antennas in a dynamic manner. However, the work presented in this paper takes advantage of the unique features of active ICs to enable the on-demand modification of not only the phase but also of the amplitude of each individual antenna element, which allows users a much greater control of the beamforming pattern (through the use of more complex modulation schemes^11^ and “on-the-fly” flex-compensation for conformal implementation^12^) as well as modularity to change “on demand” the physical aperture size of the array to suit different applications.

In this work the benefit of removable elements, modularity, massive scalability and flexibility are combined into one system. This creates a system which is easy to implement and can not only be realized in future 5G/IoT networks but also in future wearable and Reconfigurable Intelligent Surfaces (RIS) applications. These devices can be built and conformed onto various surfaces to alter the wireless channel^13^ and requires ubitquitous placement of the RIS’ across various environments, which can benefit from flexible and adaptive systems. Unlike the traditionally defined RIS structures described in^14,15^ using meta-surfaces, the RIS described in this work utilizes active devices, such as with active ICs, which allows reconfigurability with the added benefit of flexibility and conformity allowing it to be attached to any kind of surface.

### Proposed tiling solution

**Figure 2:**
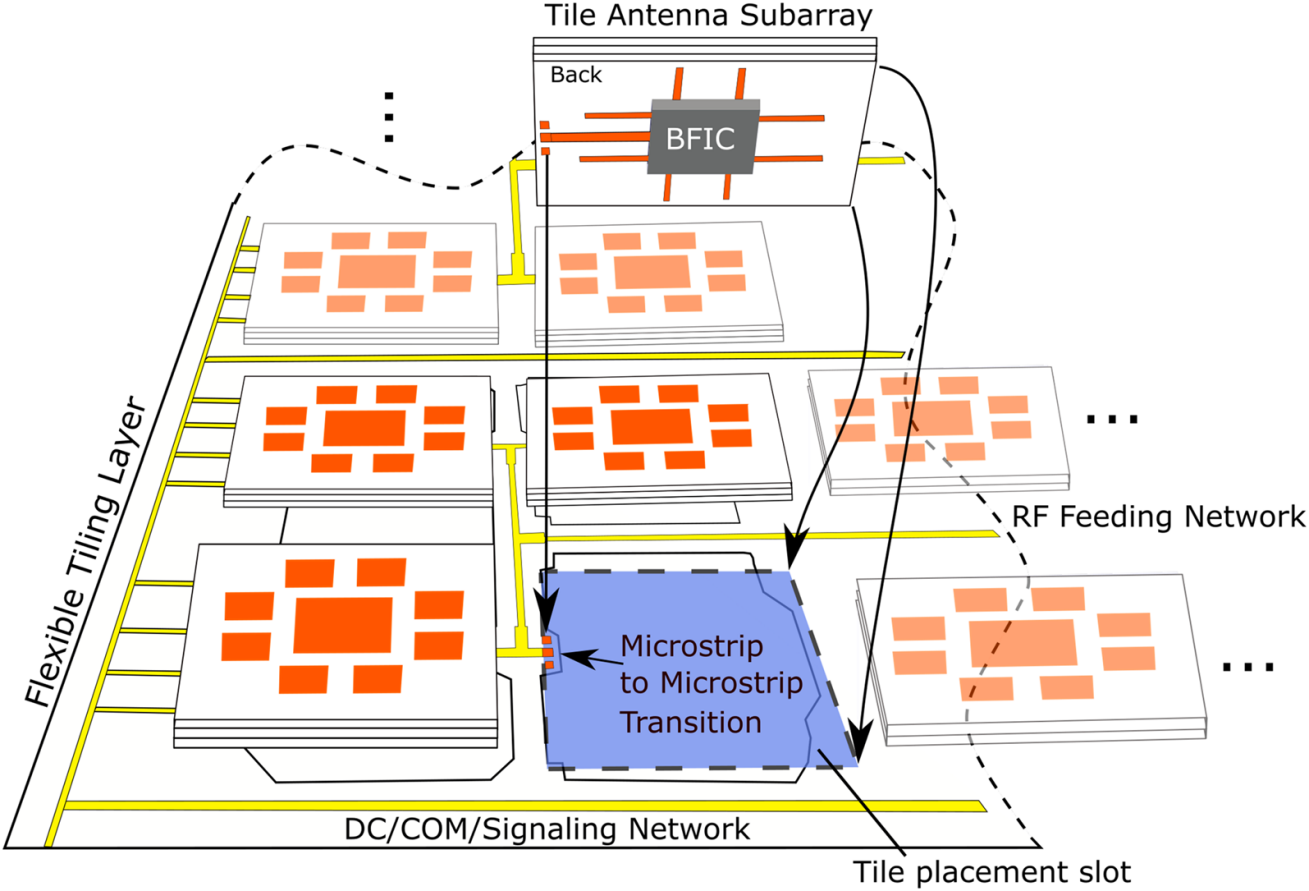
Proposed massively scalable multi-tile (“modular”) antenna array implementation. Every tile contains an antenna “subarray” along with a beamformer IC (BFIC). The tile then can be assembled onto a tiling layer, which includes the feeding, RF, DC and communication lines to enable arbitrarily large arrays.

In this work, the flexible, conformal tile architecture consists of two parts, an arbitrary number of tiles, each one of which includes an antenna subarray and an integrated beamforming IC, and an underlying tiling layer to seemlessly interconnect the tiles into very large antenna arrays and MIMOs. Not only is this implementation lower cost than large phased array implementations, but it is integrated onto a flexible base substrate, allowing the tiles to conform to various surfaces. Each individual tile consists of one beamforming IC (BFIC) integrated with 8 antenna elements, on a small scale PCB substrate. Rather than having to fabricate multiple large phased arrays of varying sizes, each tile is identical to each other taking advantage of economies of scale. This can be assembled in a modular fashion to implement larger arrays as the situation desires, in which only a low cost flexible substrate is required. The simplicity of the design makes it a unique approach to very large on-demand phased arrays, RIS and massive MIMOs implementations as this approach simplifies the array architecture by separating the RF feeding network and the antenna elements, allowing designers to focus on the implementation of the array rather than the layout and antenna structure design. Not only is this architecture easy to assemble, it is also easy to repair “on-the-fly” as tiles can be replaced in case certain tiles becomes damaged. A more detailed diagram is shown in Fig. 2 demonstrating the tiles and the tiling layer integration. In this work, a proof-of-concept prototype of a $2 \times 2$ tile-based configuration, “8 element subarray”-tile configuration realizing a 32 antenna element antenna array onto a flexible tiling layer is presented and measured at typical 5G millimeter-wave frequencies. Each individual tile consists of an 8-element subarray along with an integrated beamforming IC (BFIC) on a small scale PCB substrate and very good performance is demonstrated for planar and bent topologies typical for practical 5G/B5G applications.

## Proof-of-concept demonstrator design

A commercially available Ka-Band BFIC TX chip from Anokiwave operating from 27.5 to 30 GHz was chosen as the BFIC for the proof-of-concept $2\times 2$ tile demonstrator. The chip features 8 output antenna ports with 12 dBm saturated power output, each capable of 5-bit phase shifting and variable gain control (11.25° LSB, 0.5dB LSB respectively), controlled using SPI protocol. The QFN packaged chip measures $6 \times 6$ mm. The design must be kept symmetrical, with the BFIC in the center of the tile. In this manner, it can be assured that the tiles can be arrayed without gaps in the antenna array ensuring uniformity. With the BFIC chosen, focus can be shifted to the antenna element design.

**Figure 3:**
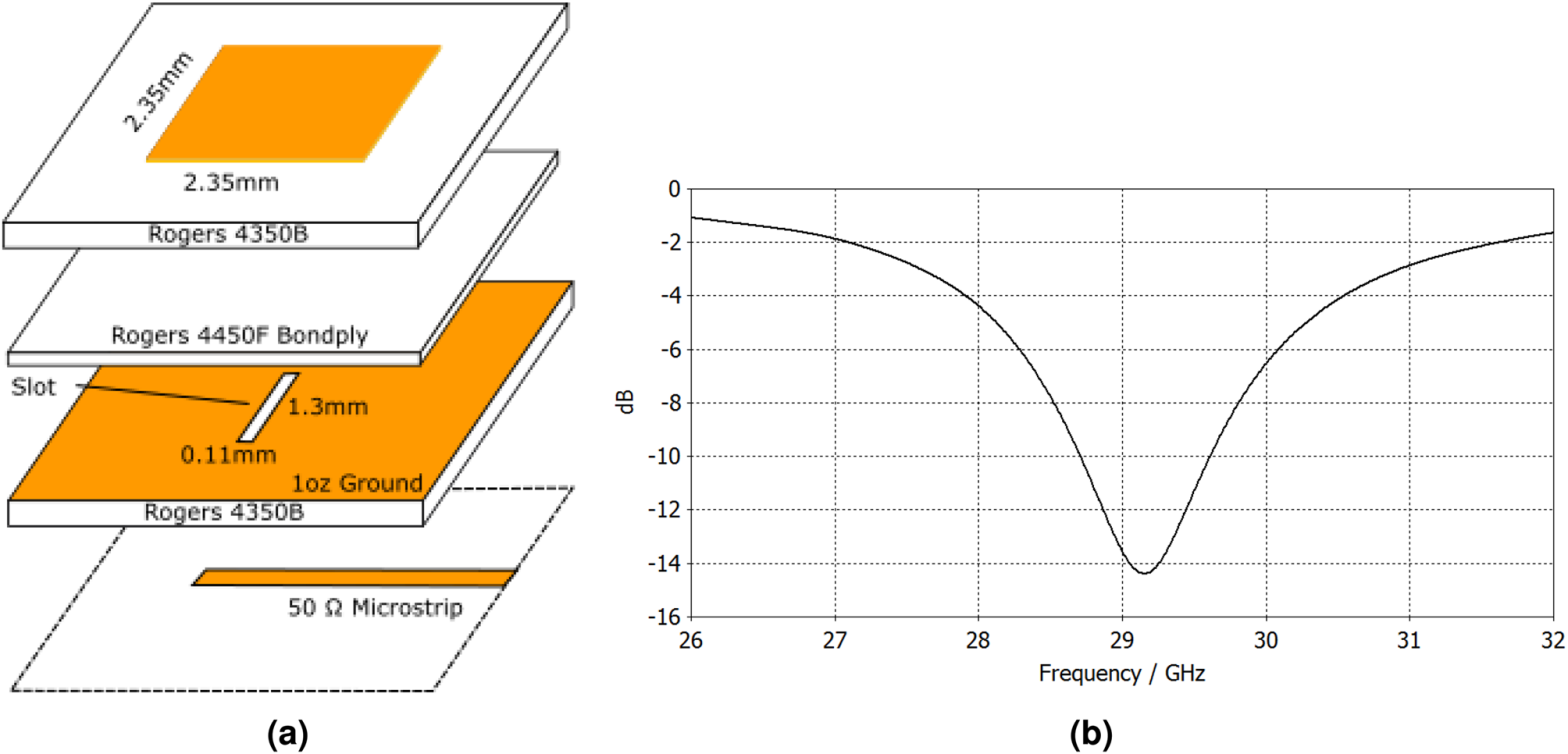
(**a**) Stackup diagram of the aperture coupled patch antenna used in tile design. (**b**) S11 results for the patch antenna targeting 29 GHz.

**Figure 4:**
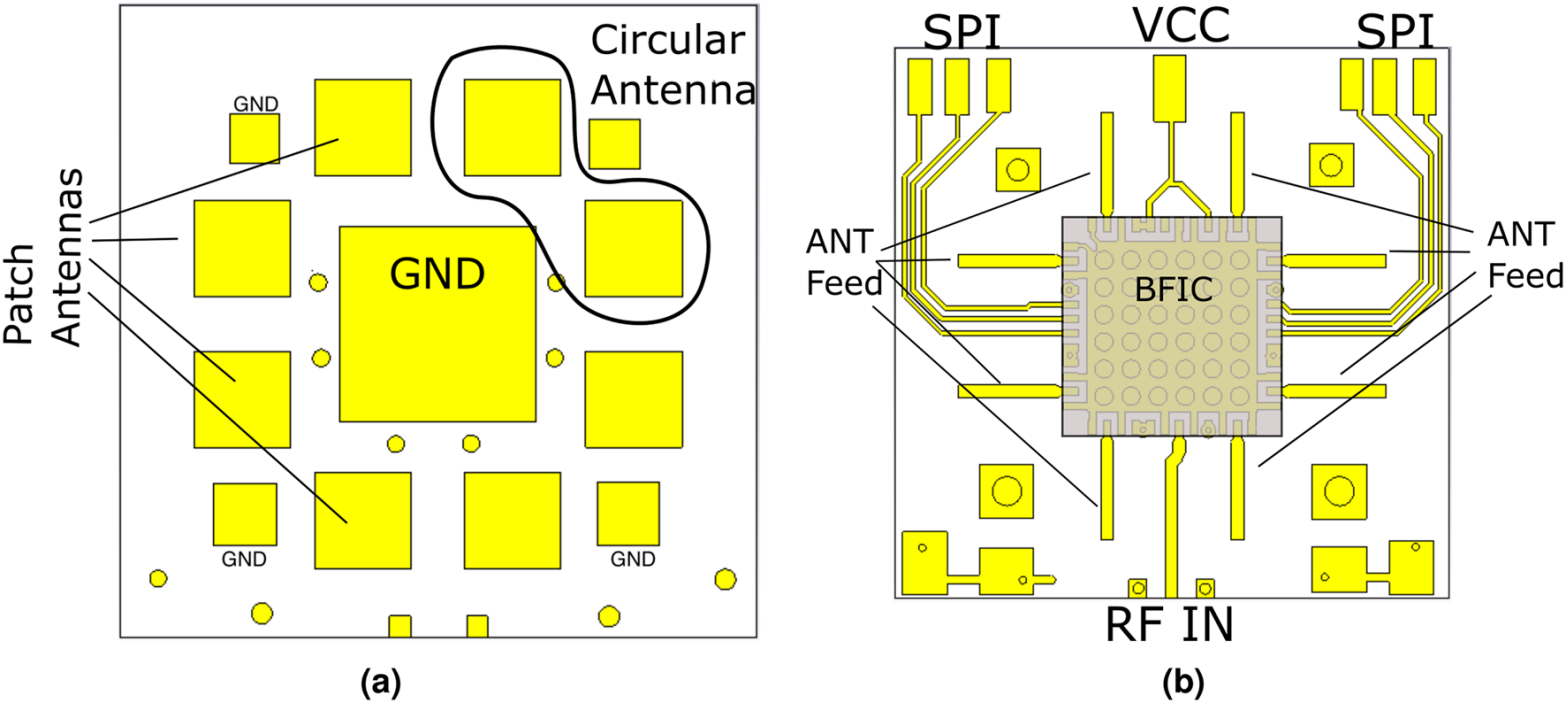
(**a**) Antenna subarray side (top side of the tile), (**b**) Beamforming IC (BFIC) and RF/DC/Digital contacts (Bottom side of the Tile). In the top-side, the patch antenna subarray elements are seen, with one circular polarized (CP) element circled as it is effectively formed by two perpendicularly linearly polarized patches with 90° phase shift. Additionally, a large thermal ground plane is also present for heat management, and some ground vias are present for extra grounding. The bottom side includes the QFN footprint for the BFIC as well as the pads for the RF, SPI and DC connections, are meant to be soldered/attached to the tiling layer.

**Figure 5:**
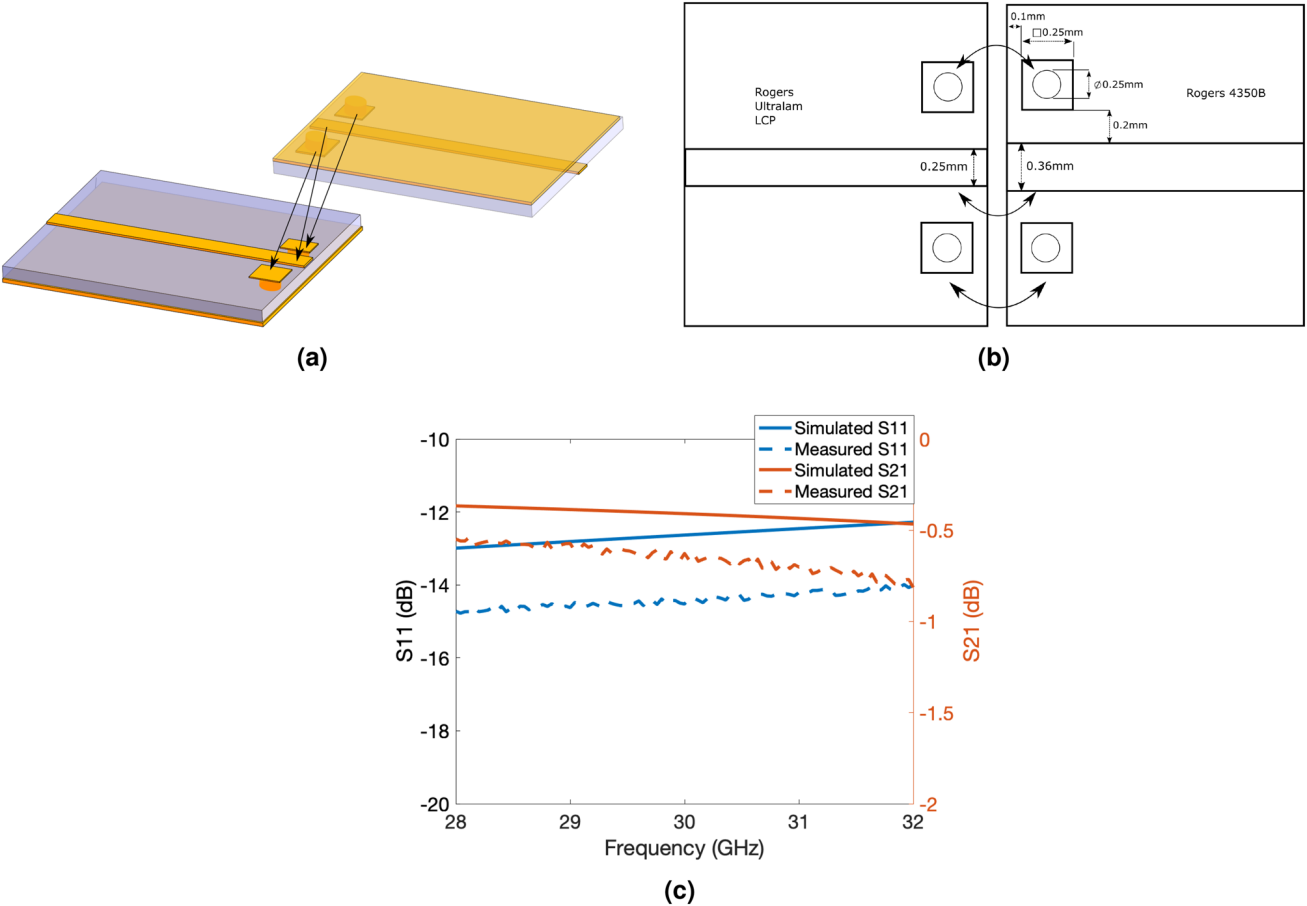
(**a**)(Tiling layer) Microstrip-to-(Tile) Microstrip transition. The connections are soldered/attached together connected via the arrows. (**b**) Dimensions of a transition between a microstrip on RO4350B and LCP. (**c**) S-Parameters of the transition over the 28–32 GHz band.

**Figure 6:**
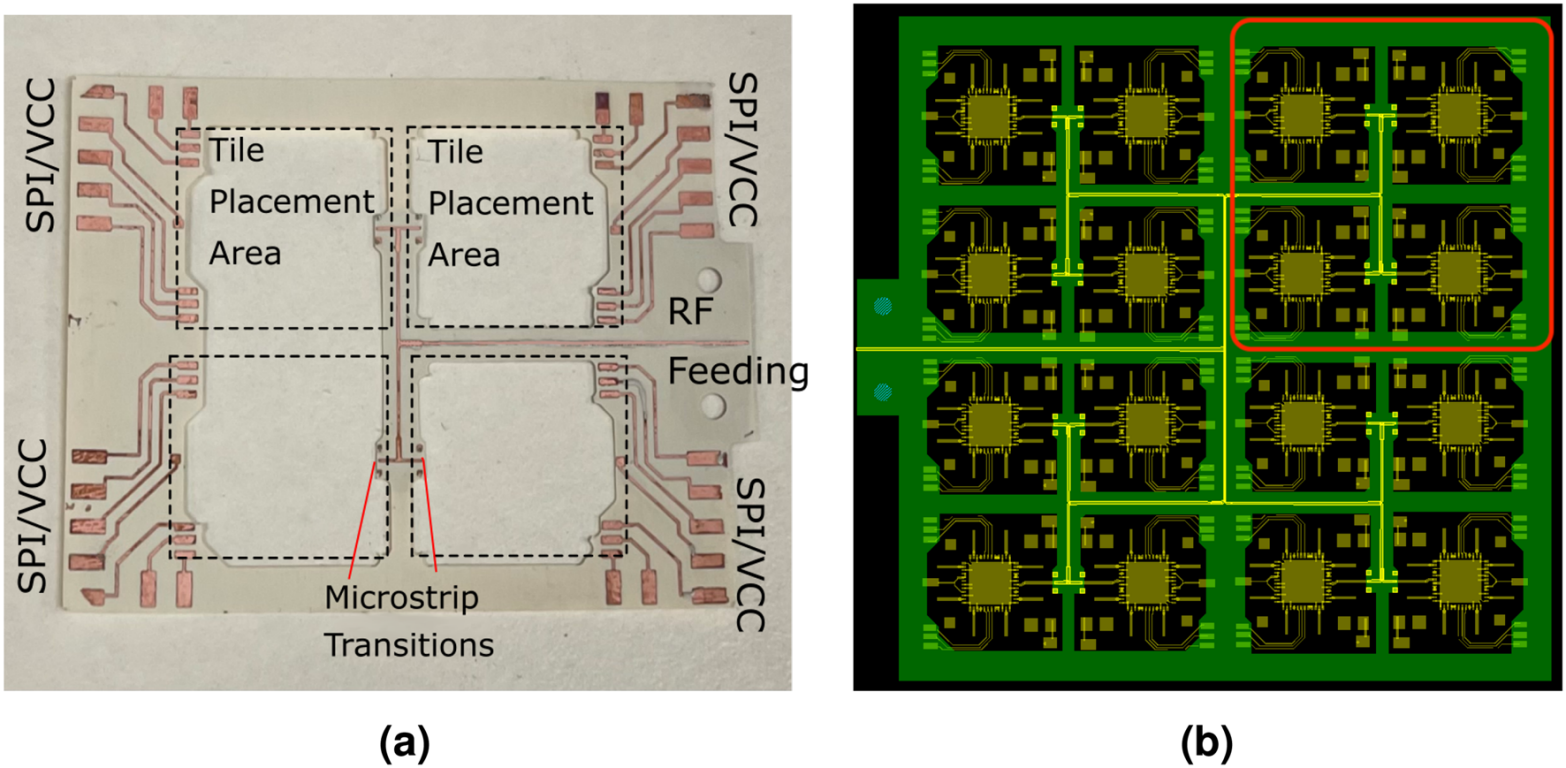
(**a**) A tiling layer for the proof-of-concept $2 \times 2$ tiled array with the SPI DC and RF lines. The pads on the outer edges are spaced for standard 2.54 mm header pins. (**b**) A proposed $4\times 4$, 128 element array tiling scheme. The circled portion of this image is one $2\times 2$ tile configuration similar to the one shown in (**a**). These structures can therefore be infinitely repeatable and massively scalable. DC and SPI lines omitted for brevity.

### Tile and antenna element design

Various antenna elements were initially considered but an aperture coupled patch antennas were chosen as the tile subarray elements for proof-of-concept demonstrations. Two common feeding mechanisms for patch antennas are microstrip and probed feeding^4,16^, however these were not chosen for several factors. The aperture coupled patch allows for a cleaner design, with antenna and the digital and DC signals separated from each other on different layers as compared to a microstrip design. Additionally, the aperture coupled antenna designs don’t require vias, unlike the probed feed. At typical 5G/B5G mmWave frequencies, vias are difficult to realize, and proper dimension controls are needed. Additionally improperly sized vias can lead to high losses and impedance mismatch reflections for RF signals^17,18^, and the need for blind and/or buried vias adds cost to the fabrication. Thus the aperture coupled patch was chosen, shown in Fig. 3a, b. prototype antenna element was built from two Rogers 6.6 mil RO4350B ($\epsilon _r = 3.66, tan\delta = 0.0037$) cores with a 4 mil RO4450F bondply ($\epsilon _r = 3.52, tan\delta = 0.004$) in between comprised of only 4 copper layers. These Rogers materials have demonstrated good performance in K-Band aperature coupled antenna arrays^19^. The prototype patch antenna had a design center frequency of 29GHz to target mmWave 5G (n257) bands as well as Ka-Band SATCOM applications. The antenna element can be made more broadband by utilizing wideband antenna elements demonstrated in^20^ which cover all of the expected 5G mmWave spectrum from 24 to 40 GHz. These aperature coupled patch antenna elements were integrated in subarrays on each tile effectively enabling very large antenna arrays and massive MIMO configurations.

The effective aperture efficiency of a single tile was calculated to be 37% efficient according to Eq. 1:

1 $$\begin{aligned} {e_{a} = \frac{\lambda ^{2}}{4\pi A_{phys}}G_{T} } \end{aligned}$$

where $A_{phys}$ and $G_{T}$ are the physical aperture size and the gain of the tile element respectively. Each tile element has 9.7 dBi gain with an area of 14.5mm x 14.5mm at 29GHz. This is lower than typical high efficiency aperture microstrip antennas such as the ones demonstrated in^21,22^ which can achieve >60% aperture efficiency. The loss in efficiency is mainly due to the size of the BFIC and the need for the large ground for thermal management. As increasingly more BFICs become commercialized, newer BFICs can have drastically smaller footprints sizes thus the efficiency can be increased closer the conventional values around 60%.

The antenna elements, and thus the antenna tiles, were fabricated with rigid materials as opposed to flexible materials like LCP or polyimide. This is because it is difficult to obtain thick flexible substrates in order for the antennas to have sufficient bandwidth to cover significant portions of 5G mmWave bands. Thus, the RO4350B substrate was chosen. Additionally, identical material cores, with layer symmetry (RO4350B, RO4450F, RO4350B) were chosen due to the need for identical coefficient of thermal expansion (CTE) matching so that soldering doesn’t warp the substrate making the tiles unusable. These factors combined, makes the tiles themselves a rigid structure. Without loss of generality and for proof-of-concept demonstration purposes, 8 antenna elements were placed onto a single tile, with each antenna connected to one of the 8 output ports on the BFIC. This gives the BFIC control of each individual antenna element and thus allows for greater control of the phased array direction. The antennas are arranged in a configuration with 4 in linear vertical polarization and 4 in linear horizontal polarizations (2 top, 2 left, 2 right and 2 bottom). With these antenna elements in this configuration, a CP antenna array can be generated by lagging either the horizontal or vertical polarization with each other by 90° ^23^, which is easily achieved using the BFIC internal phase shifter. The 8 elements were placed in this configuration in order for all of them to be as close to the output port pins of the chip as possible, and the antenna element feed lines are kept straight, shown in Fig. 4b, to minimize any losses. With the BFIC measuring $6\times 6$ mm, patch antenna elements measuring $2.35\times 2.35$ mm, as well as sufficient substrate area to allow the patch antennas to radiate, a single tile itself measures $14.5 \times 14.5 \times 0.52$ mm.

Each tile includes multiple contact points which align to the pads placed on the tiling layer. On the top side (the antenna side Fig. 4a), is the antenna elements with a grounding pad, while on the other side (the chip side 4b), lies the BFIC and the RF transmission lines feeding the antenna elements and the SPI and VCC elements. The grounding pad is necessary to ensure an effective, low inductance RF ground with multiple vias as well as good heat dissipation, as the BFIC consumes 1.4 W under P1 dB conditions.

### Flexible tiling layer design

The flexible tiling layer is built on a single Rogers liquid crystal polymer (LCP) ULTRALAM substrate. LCP is a high performance material that is flexible and resistant to moisture and temperature making it a suitable material for outdoor use, such as in outdoors 5G basestations. LCP has been characterized and demonstrates excellent low loss RF properties at least from 30 to 110 GHz^24^ covering most 5G/B5G frequency bands. A 4 mil LCP layer was utilized for the proof-of-concept tiling layer substrate. The LCP was double sided patterned with one RF, DC and SPI distribution layer, while the other side pattern allowed for the realization of the ground structure under microstrip line only, in order to enhance the flexibility. This was done just to enhance the flexibility of the structure however it is not required. Holes were cut around the center of the tiling layer substrate to allow for accurate mounting of the tiles without blocking their subarray radiation characteristics. The mounting pads themselves act as alignment markers to align the antenna tiles to their respective designed positions.

**Figure 7:**
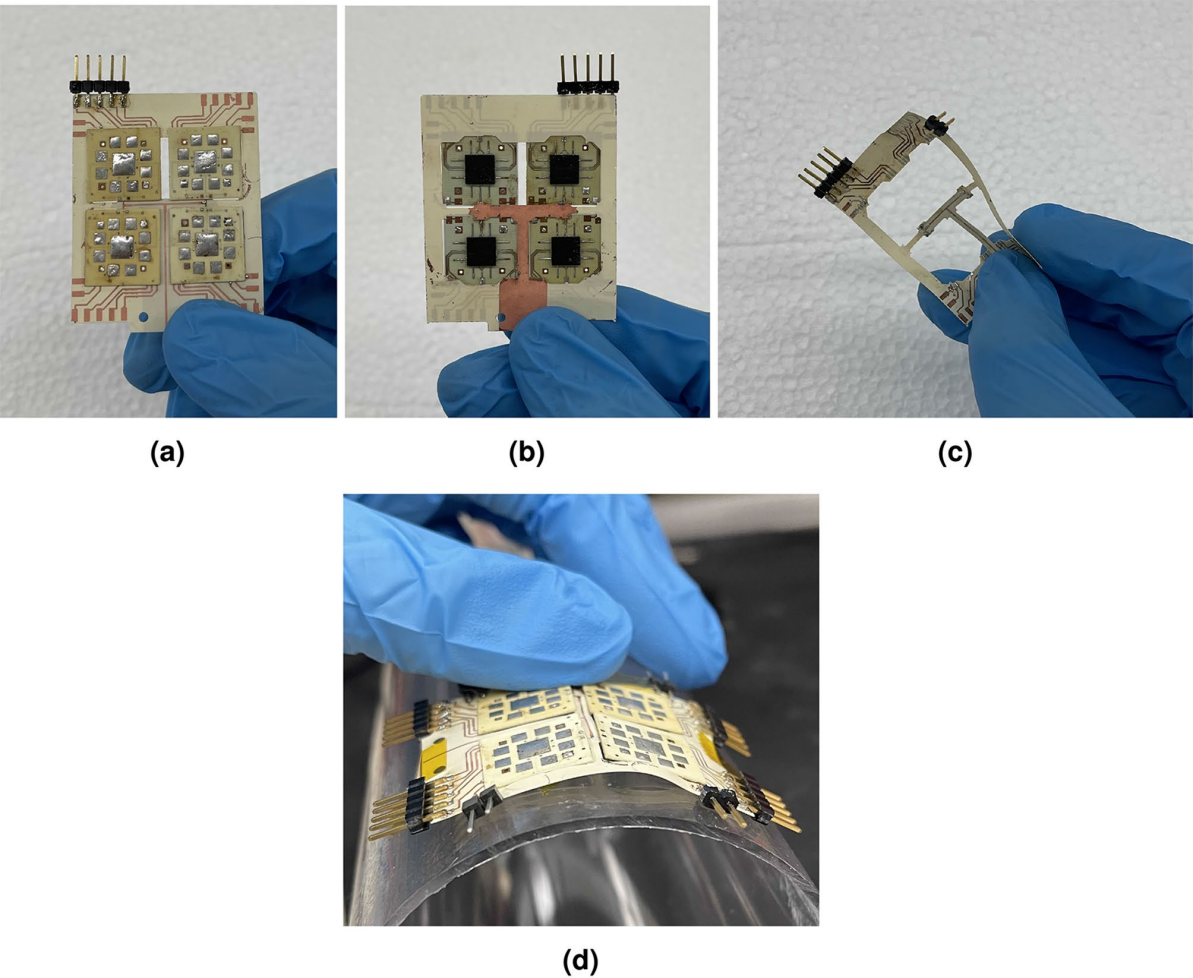
(**a**) Front, antenna side and (**b**) Back, chip side. (**c**) Flexible tiling layer being bend in hand and (**d**) being conformed to a 3.5 cm radius cylinder. The flexible tiling layer combined with the tiles facilitates the flexibility. The system is naturally flat, so to conform to a curvature, it needs to be forced to the curve, either using adhesive or by hand shown here.

A critical component is the tiling layer microstrip to tile microstrip transition shown in Fig. 5. Other interconnecting approaches have been previously demonstrated in literature^25,26^, utilizing coupled sections or vias. However, the proposed tile-based massively scalable MIMO and phased-arrays topologies are designed to be modular, thus an approach is needed which allows them to be easily assembled or disassembled on-demand. A straightfoward approach was implemented where one microstrip was flipped onto the other with two grounding pins on either side of the microstrip connecting the ground together, shown in Fig. 5a. It was observed in simulation that the performance characteristics, insertion and return loss, are primarily controlled by the distance of the gap between the via pads and microstrip line, and the offset from the edge. An optimized transitions with its parameters is shown in Fig. 5b. This transition design has a low insertion loss of less than 0.5dB across the simulated frequency range of 28–32 GHz Fig. 5c. These two halves of the transitions are soldered to each other and provide additional structural support to the tiles under bending conditions. The manual assembly and soldering of these transitions account for the additional losses that were not seen in the simulation. The other pads for SPI and DC are also soldered together. This allows easy assembly and removal, utilizing only a hot air gun to reflow the soldered joints. A corporate feeding network was designed for the 4 tiles along with the fan-out of the SPI and DC lines. The 50 ohm transmission lines with the tile terminations are joined together and matched to another 50 ohm transmission line with a $\lambda /4$ impedance transformer, centered at 29 GHz, with impedance $Z_{o} = \sqrt{50\cdot 25}$. This process can be repeated for larger arrays. The tiling layer for a $2\times 2$ tiled array is shown in Fig. 6a. Note that the tiling layer is not exactly symmetric, as VNA measurement setup requires a 2.92mm end-launch connector which takes up a fair amount of real estate on the tiling layer. Additionally, standard 2.54mm header pin connectors were utilized for the DC and SPI connections for easy wiring and connection to the controller. For actual implementations, the end-launch connector and header pins might not be necessary and thus the tiles can form a more symmetrical layout and can be further miniaturized. A $4\times 4$ scale-up version is shown Fig. 6b. The array is easy to scale as the 4x4 topology is simply made up of 4 $2\times 2$ sections with an extended RF corporate feeding network. These structures can therefore be infinitely repeatable, only limited by factors such as dielectric and ohmic losses of the feeding network^27^.

The antenna elements are symmetric with respect to each other, meaning that the antenna element on the opposite side, is 180°phase shifted, effectively being out-of-phase with the antisymmetrical element, an effect that is easily corrected by programming the BFIC to provide an 180°phase adjustment leading to all in-phase elements. Because of the symmetry of the tiles, the chip needs to be in the middle. A single 8 element proof-of-concept tile itself measures $14.5\times 14.5 \times 0.52$ mm. In the $2\times 2$ array, the tiles are spaced 2 mm apart. Its widely known that the array spacing should be kept small to avoid grating lobes^28^, but in this work, the extra spacing is necessary so that the tiles do not cross over and interfere with the microstrip feed line on the tiling layer, hence the 2mm spacing distance. This can be mitigated in future works by utilizing a stripline approach, such that the transmission lines are covered with copper, thus isolating the transmission line from the antenna elements. Striplines, however, are more difficult to fabricate than microstrip and requires more dielectric material which introduces cost and vias which could introduce more loss, thus a simpler microstrip approach was taken for this work.

**Figure 8:**
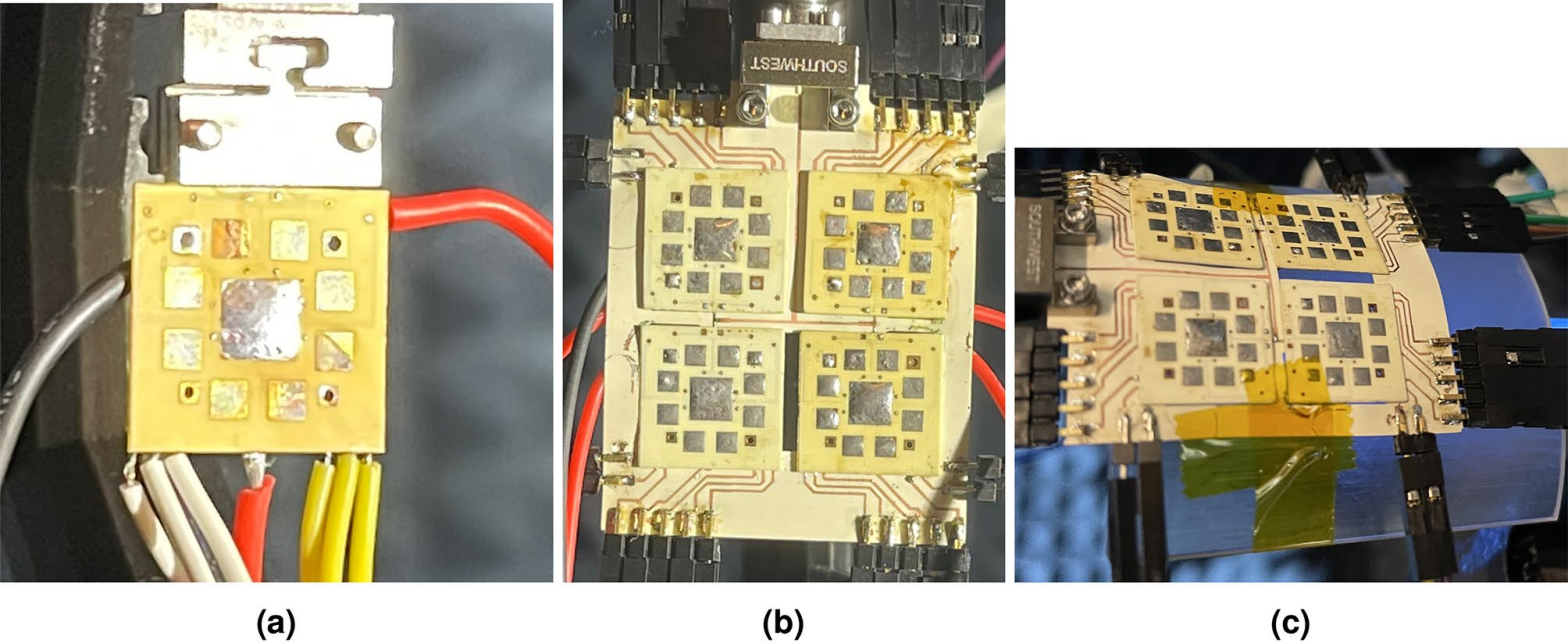
Assembled tiles with wires attached for power and SPI. (**a**) Single tile implementation and (**b**) multi ($2\times 2$ 32 element) implementation. (**c**) Additionally, the tiles were measured while conformed to a 3.5 cm bend radius.

In Fig. 7a, b the assembled $2\times 2$ “8 element subarray” tiles proof-of-concept 32 antenna array prototype is shown in front and back sides. In Fig. 7b, the backside shows that the copper is left behind only underneath the microstrip transmission lines. Since the LCP is only 4 mils thick, substrate is very flexible as shown in Fig. 7c. The fully assembled $2\times 2$ array is seen in Fig. 7d being conformed around a cylindrical bend with radius 3.5 cm, with no delamination or cracks appearing.

**Figure 9:**
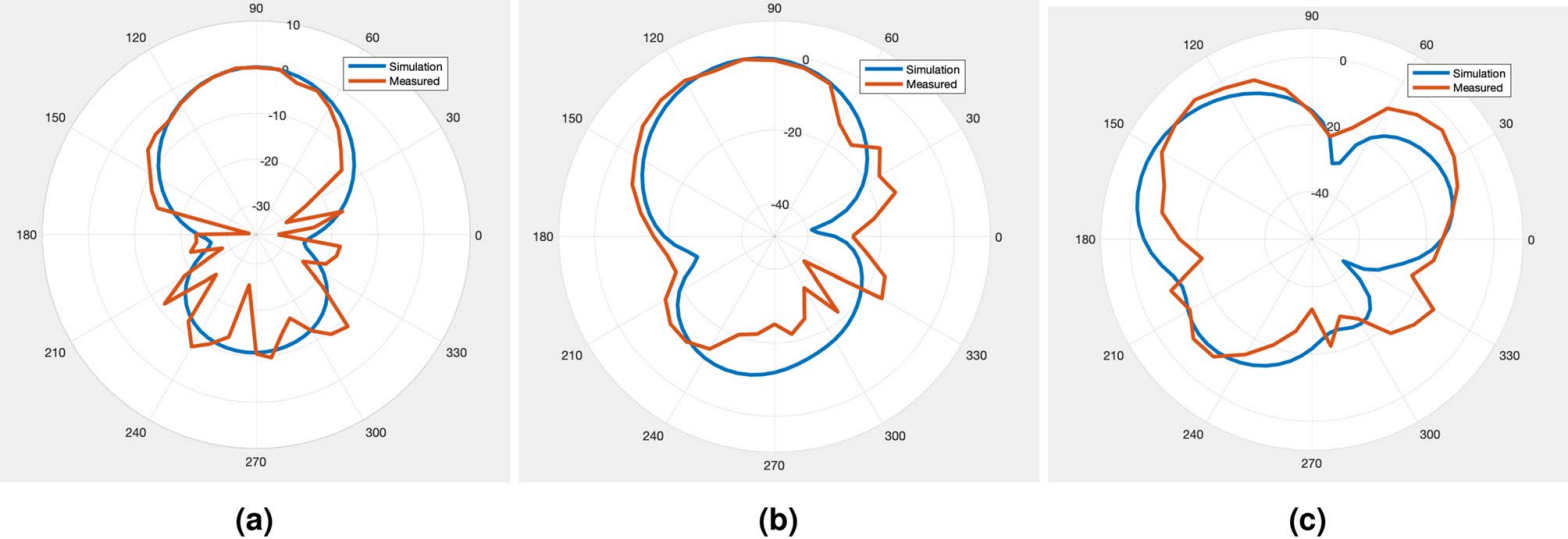
Single Tile measurement results demonstrating both measured and simulated normalized gain patterns across the H-Plane of the prototype $2\times 2$ tiled array. (**a**) equal phased elements, (**b**) 55° (**c**) 123° progressive phase shifting.

## Measurements

Both single tile and multi-tile prototype implementations were built and measured. For the single tile no tiling layer was required but the tiling layer was required and demonstrated in the 2x2 tile array. The control software was written in MATLAB to control the SPI using a NI USB-8452 I$^{2}$C/SPI interface.

**Figure 10:**
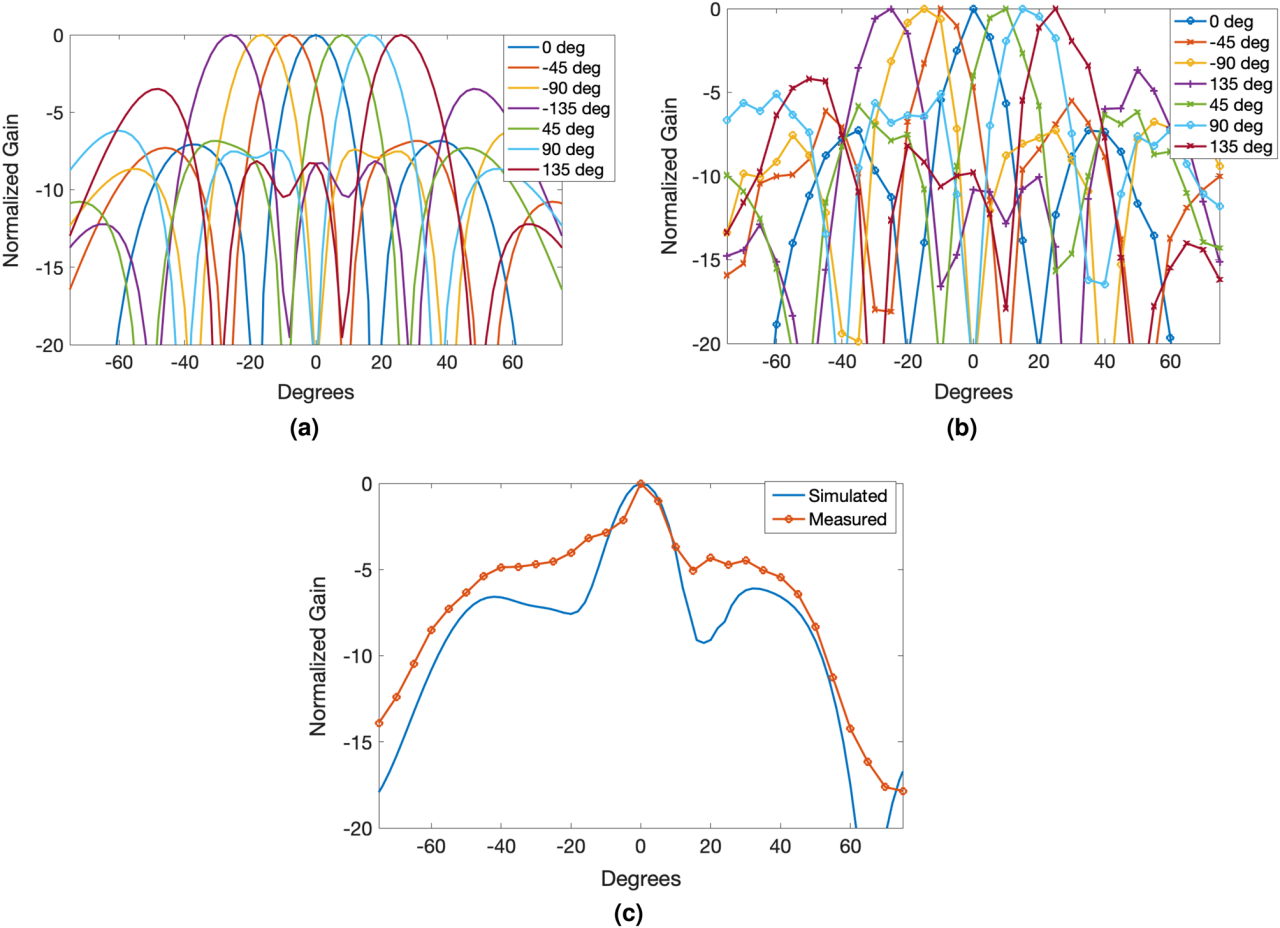
(**a**) Simulated measurement results for progressive phase shift of $-135$ to $+135$° between CP elements. (**b**) Measured results from the same progressive phase shift values demonstrates good agreement with the simulated results. (**c**) Measurement results of a $2\times 2$ array bent on a 3.5 cm radius compared to simulation.

In Fig. 8 both the single and multi-tile prototype implementation were shown in the anechoic chamber. The measurement results for the gain pattern for the single tile can be seen in Fig. 9 and multi-tile in Fig. 10. To realize electronic beam scanning capabilities, the antenna elements require a progressive phase shift. Since the tiles operate in circular polarization, the two antennas, which are 90° offset from each other, can be considered as a single circular polarized antenna. This way, in each direction of scanning (horizontal and vertical) there are 4 “equivalent” antenna elements per tile. For demonstration purposes, the 2 $2\times 2$ tiled array was steered in both directions in the horizontal axis using a progressive phase shift of additional 45° per measurement up to 135°. The measurement data shows some minor deviations and “noise”. This is most likely due to the fact that because the substrate is flexible, the substrate could be resting in a non-planar state unlike a rigid substrate. These micro-distortions can cause minor fluctuations in the measurement setup as well as introduce small phase distortions since the tiles are not in their optimal locations. Future works can utilize pattern prediction techniques to mitigate these effects of flexible substrates^12,29,30^.

The patterns shown in Fig. 10 show that for the measured case, a 4.5 db side lobe is observed at high steering angle. This can be reduced by decreasing the spacing separating the tiles, and converting the microstrip feedline to a stripline topology.

To demonstrate the very good flexibility characteristics of the proposed tile-based modular approach, the $2\times 2$ tiles prototype was conformed onto a 3.5cm radius. The expected behavior is that the bending will flatten the gain pattern and “fan-out” the pattern, which is demonstrated in the simulation. The antenna elements all have equal phasing, ie. full broadside pattern. This was confirmed in measurement, as the antenna gain pattern follows the simulated result. However, the gain pattern features some minor distortions compared to the simulations probably due to the uneven planarity of the array. On the backside, the mounted BFIC creates an uneven surface making it difficult to fully conform to flat surfaces, which leads to phase offsets and thus deviations in the pattern. These factors stress the importance of utilizing pattern prediction techniques mentioned above to mitigate these deviations as the flexibility of the array can also lead to some unwanted effects.

## Conclusion

A novel tile-based approach enabling the modular realization of massively scalable MIMO and phased arrays for 5G/B5G millimeter-wave smart skins and large-area RIS for Smart Cities and IoT applications was introduced in this paper. The unique benefits of the proposed tile approach utilizes the fact that tiles of identical sizes can be manufactured in large quantities rather than having to realize arrays of multiple sizes in order to serve various application needs and user capacity coverage areas. The trade-off of this type of design is the utilization of additional manufacturing steps, such as an increase in assembly time, and additional transmission losses due to the need for a transition component.

In summary, a proof-of-concept 29 GHz $2\times 2$ tiles based 32 element antenna array was fabricated and measured and demonstrates $+/-$ 30beamsteering capability featuring no performance degradation when it is wrapped around a 3.5 cm radius curvature. This topology can be easily scaled up to massively large arrays by simply adding more tiles and extending the feeding network on the mounting tiling layer. The tiles are assembled onto a single flexible tiling substrate which interconnects the RF, DC and digital traces, allowing for the easy realization of on-demand very large antenna arrays and massive MIMOs on virtually any practical conformal platform for frequencies up to sub-THz frequency range. The tiling topology used in this work in conjunction with active BFICs allows for more complex modulations and beamforming control and with the combination of flexible and conformal capabilities, enables this system to greatly enhance not only 5G and beyond RIS’ but also for smart cities, autonomous cars and smart skins applications.

## References

[1] Hong W (2017). Multibeam antenna technologies for 5g wireless communications. IEEE Trans. Antennas Propag..

[2] Hur S (2013). Millimeter wave beamforming for wireless backhaul and access in small cell networks. IEEE Trans. Commun..

[3] Morrison, G.D., McLachlan, A. D. & Kinghorn, A.M. “Tile”-based airborne phased array radar systems. In *International Conference on Radar Systems (Radar 2017)*, 1–4 (2017).

[4] Lyon, R. W. *et al.* Active electronically scanned tiled array antenna. In *2013 IEEE International Symposium on Phased Array Systems and Technology*, 160–164 (2013).

[5] Shahramian S, Holyoak MJ, Singh A, Baeyens Y (2019). A fully integrated 384-element, 16-tile, $$w$$ -band phased array with self-alignment and self-test. IEEE J. Solid-State Circuits.

[6] Anselmi, N., Rocca, P. & Massa, A. Modular phased array design through a tile-dimension tapering approach. In *2019 IEEE International Symposium on Antennas and Propagation and USNC-URSI Radio Science Meeting*, 1–2 (2019).

[7] Hashemi MRM (2019). A flexible phased array system with low areal mass density. Nat. Electron..

[8] Ren, L., Lu, B., Lu, F. & Shu, Y. Modular and scalable millimeter-wave patch array antenna for 5g mimo and beamforming. In *2020 50th European Microwave Conference (EuMC)*, 336–339 (2021).

[9] Akbar, F. & Mortazawi, A. A k-band low-complexity modular scalable wide-scan phased array. In *2020 IEEE/MTT-S International Microwave Symposium (IMS)*, 1227–1230 (2020).

[10] Shlezinger N, Alexandropoulos GC, Imani MF, Eldar YC, Smith DR (2021). Dynamic metasurface antennas for 6g extreme massive mimo communications. IEEE Wirel. Commun..

[11] Kibaroglu K, Sayginer M, Phelps T, Rebeiz GM (2018). A 64-element 28-ghz phased-array transceiver with 52-dbm eirp and 8–12-gb/s 5g link at 300 meters without any calibration. IEEE Trans. Microw. Theory Tech..

[12] He, X. & Tentzeris, M. M. In-package additively manufactured sensors for bend prediction and calibration of flexible phased arrays and flexible hybrid electronics. In *2021 IEEE MTT-S International Microwave Symposium (IMS)* (2021).

[13] ElMossallamy MA (2020). Reconfigurable intelligent surfaces for wireless communications: principles, challenges, and opportunities. IEEE Trans. Cognit. Commun. Netw..

[14] Huang C, Zappone A, Alexandropoulos GC, Debbah M, Yuen C (2019). Reconfigurable intelligent surfaces for energy efficiency in wireless communication. IEEE Trans. Wireless Commun..

[15] Alexandropoulos GC, Shlezinger N, del Hougne P (2021). Reconfigurable intelligent surfaces for rich scattering wireless communications: recent experiments, challenges, and opportunities. IEEE Commun. Mag..

[16] Hertleer C, Tronquo A, Rogier H, Vallozzi L, Van Langenhove L (2007). Aperture-coupled patch antenna for integration into wearable textile systems. IEEE Antennas Wirel. Propag. Lett..

[17] Watanabe AO, Ito H, Markondeya RP, Tummala RR, Swaminathan M (2020). Low-loss impedance-matched sub-25-m vias in 3-d millimeter-wave packages. IEEE Trans. Compon. Packag. Manuf. Technol..

[18] Yu, C. H. *et al.* High performance, high density rdl for advanced packaging. In *2018 IEEE 68th Electronic Components and Technology Conference (ECTC)*, 587–593 (2018).

[19] Yi Z (2019). A wide-angle beam scanning antenna in e-plane for k-band radar sensor. IEEE Access.

[20] Lin T-H (2020). Broadband and miniaturized antenna-in-package (aip) design for 5g applications. IEEE Antennas Wirel. Propag. Lett..

[21] Guo J, Liao S, Xue Q, Xiao S (2017). Planar aperture antenna with high gain and high aperture efficiency for 60-ghz applications. IEEE Trans. Antennas Propag..

[22] Jang TH (2016). A wideband aperture efficient 60-ghz series-fed e-shaped patch antenna array with copolarized parasitic patches. IEEE Trans. Antennas Propag..

[23] Huang J (1986). A technique for an array to generate circular polarization with linearly polarized elements. IEEE Trans. Antennas Propag..

[24] Thompson D (2004). Characterization of liquid crystal polymer (lcp) material and transmission lines on lcp substrates from 30 to 110 ghz. IEEE Trans. Microw. Theory Tech..

[25] He G, Gao X, Zhang R (2021). Impact analysis and calibration methods of excitation errors for phased array antennas. IEEE Access.

[26] Tsai C-C, Cheng Y-S, Huang T-Y, Hsu YA, Wu R-B (2011). Design of microstrip-to-microstrip via transition in multilayered ltcc for frequencies up to 67 ghz. IEEE Trans. Compon. Packag. Manuf. Technol..

[27] Levine E, Malamud G, Shtrikman S, Treves D (1989). A study of microstrip array antennas with the feed network. IEEE Trans. Antennas Propag..

[28] Balanis CA (2015). Antenna Theory: Analysis and Design.

[29] Fikes, A. C., Safaripour, A., Bohn, F., Abiri, B. & Hajimiri, A. Flexible, conformal phased arrays with dynamic array shape self-calibration. In *2019 IEEE MTT-S International Microwave Symposium (IMS)*, 1458–1461 (2019).

[30] Braaten BD (2013). A self-adapting flexible (selflex) antenna array for changing conformal surface applications. IEEE Trans. Antennas Propag..

